# Cephaline in Amoebiasis

**Published:** 1917-02

**Authors:** 


### ABSTRACTS

Have you ever felt that this department of the BUFFALO MEDICAL JOURNAL was lacking in abstracts of your specialty, or that such as were published lacked the expert judgement which you yourself could have exercised in selection and preparation? If not, you are more charitable than the editor has been to himself. If so, will you assist in abstracting from a few journals, and thus make this department more representative and more valuable? Incidentally, there are few forms of study more helpful to the worker than this.

**Cephaline in Amoebiasis.** Sidney K. Simon, New Orleans, N. O. Med. Jour., Dec. 1916. This new alkaloid of ipecac has at least an equal amoebicidal (amoebectanic?) action on free-living entamoebae as compared with emetine, promises greater success with encysted organisms, produces less diarrhoea but more nausea and vomiting and greater irritation and pain when injected subcutaneously. No evidences of toxaemia were noticed following the small therapeutic doses. A combination with emetine, for hypodermatic injection, is suggested.

